# Parental and child adjustment to amyotrophic lateral sclerosis: transformations, struggles and needs

**DOI:** 10.1186/s40359-022-00780-1

**Published:** 2022-03-17

**Authors:** Marion Sommers-Spijkerman, Neele Rave, Esther Kruitwagen-van Reenen, Johanna M. A. Visser-Meily, Melinda S. Kavanaugh, Anita Beelen

**Affiliations:** 1 Department of Rehabilitation, Physical Therapy Science and Sports, Brain Center, University Medical Center Utrecht, Utrecht, The Netherlands; 2 Center of Excellence for Rehabilitation Medicine, Brain Center, University Medical Center Utrecht, and De Hoogstraat Rehabilitation, Utrecht, The Netherlands; 3 Helen Bader School of Social Welfare, University of Wisconsin-Milwaukee, Milwaukee, WI USA

**Keywords:** Amyotrophic lateral sclerosis, Family, Parenting, Support needs, Qualitative research

## Abstract

**Background:**

Amyotrophic lateral sclerosis (ALS), progressive muscular atrophy (PMA) and primary lateral sclerosis (PLS), together referred to as ALS, are life-limiting diagnoses affecting not only patients but also the families surrounding them, especially when dependent children are involved. Despite previous research highlighting the vulnerability of children in these families, they are, as yet, often overlooked in healthcare. Efforts are needed to better support children in families living with ALS, both directly and through strengthening parents in their parental role. This study sought to gain a better understanding of parental and children’s experiences, struggles and support needs in families living with ALS.

**Methods:**

Semi-structured interviews were conducted with 8 parents with ALS, 13 well parents and 15 children, together representing 17 families. Interview data were analyzed using qualitative content analysis.

**Results:**

Three major themes were identified relating to (1) ALS-related transformations in families’ homes, activities, roles and relationships, that trigger (2) distress among families, which, in turn, evokes (3) emotional, psychological, educational and practical support needs. For emotional and practical support, parents and children mainly rely on their own family and social network, whereas they seek educational and psychological support from healthcare professionals.

**Conclusions:**

Our findings imply that ALS care professionals may foster family adjustment to living with ALS, most notably through encouraging parents to engage in a dialogue with their children about the many transformations, struggles and needs imposed by ALS and teaching them how to start the dialogue.

**Supplementary Information:**

The online version contains supplementary material available at 10.1186/s40359-022-00780-1.

## Introduction

Amyotrophic lateral sclerosis (ALS), primary lateral sclerosis (PLS) and progressive muscular atrophy (PMA) are incurable diseases, characterized by progressive degeneration of upper and/or lower motor neurons [1]. Although the prognosis differs, there is considerable overlap in symptoms between these types of motor neuron diseases. All three lead to muscle atrophy, weakness and fasciculation, which negatively impacts on patients’ ability to walk, speak, swallow, and breathe [1]. As a consequence, they become increasingly dependent on care and equipment. In addition, patients may exhibit cognitive and behavioral impairments [2]. Care for those living with ALS, PMA and PLS is primarily provided by the families that surround them impacting on family functioning [3, 4]. Given the complexity of care needs and disease progression, it is critical to understand how ALS, PMA and PLS affect families, most acutely those who have children in the home.

There is a paucity of research in families living with PMA or PLS yet the few existing studies in the field of ALS suggest that having a parent with ALS makes children vulnerable in multiple respects. In a case–control study, Calvo and colleagues [5] found that children/adolescents of a parent with ALS experience significantly more anxiety and depressive symptoms and more behavioral problems compared to children with two well parents. Other research demonstrates that children in families with ALS are frequently involved in caregiving tasks, including assistance with moving around, eating, toileting, bathing and administering medications [6, 7]. Children who provide care for their seriously ill parent experience a below average quality of life and generally perceive caregiving as stressful [8].

Whereas these studies stress the importance of having adequate support systems for children of a parent with ALS, children are, as yet, often overlooked by health care professionals when it comes to providing information and support. Meanwhile parents worry about the impact of ALS on their children’s lives, are uncertain how to communicate to their children about ALS, and struggle with how to optimally support their children in dealing with the diagnosis, the physical and emotional deterioration of the parent and the prospect of parental loss [9–11], leaving children confused, questioning and without guidance. To protect children throughout the illness trajectory, it is thus not only crucial to support children themselves, but first and foremost to empower parents to support their family. To this end, we need to have a better understanding of the specific support needs of parents and children in families living with ALS.

The current literature about support needs of families living with ALS focuses primarily on care needs, i.e., needs with respect to diagnostic care, home care, counselling, assistive and adapted equipment, respite care and bereavement care [12–14], while only very little attention has been paid to the children’s and parental needs. In the present study, we sought to explore how families with children experience living with ALS, PMA or PLS, what they struggle with and what types of support they need.

## Methods

This study was not pre-registered. Findings are reported in accordance with the consolidated criteria for reporting qualitative studies (COREQ; 15). The completed COREQ checklist can be found in Additional file 1.

### Recruitment and participants

Via advertisements and/or personal contacts of ALS Centre Netherlands, patient associations and ALS care professionals, we recruited Dutch families living with ALS, PMA or PLS, i.e., families with children in which a parent is diagnosed with ALS, PMA or PLS. For the sake of readability, in the remainder of this paper, we just use the abbreviation ALS when referring to one or more types of abovementioned motor neuron diseases. Within these families, the parent with ALS, the well parent, as well as children/adolescents in the ages 12–25 years were asked to take part in an interview. Parents were eligible to participate in this study if they or their spouse/co-parent were diagnosed with ALS and had one or more children ≤ 25 years. Children/adolescents were eligible if they had a parent diagnosed with ALS and were aged between 12 and 25 years. Bereaved parents and children could also participate. Criteria for exclusion were (1) insufficient Dutch language proficiency, (2) inability to communicate verbally, (3) severe cognitive impairment, not able to make a conversation, and/or (4) unwillingness or inability to provide voluntary and informed consent to participate in an interview. Individuals interested in study participation were requested to send an email with their contact details to the first author and were subsequently approached via email or telephone.

From March 2020 to November 2020, thirty-seven interviews were conducted. Audio recording failed during one interview, leaving 36 interviews for analysis. The final sample included 8 parents with ALS, 13 well parents and 15 children/adolescents from 17 families. In those families, the patient’s diagnosis dated back between 5 months and 12 years before the interview. In two families, the parent with ALS deceased, 3 months or 8 years before the interview, respectively. Demographic and illness-related characteristics are shown in Table 1.

**Table 1:** Demographic and illness-related characteristics (N = 36)

Characteristic	Parents with ALS (*N* = 8)	Well parents (*N* = 13)	Children (*N* = 15)
*Gender, n (%)*			
Male	6 (75.0)	3 (23.1)	5 (33.4)
Female	2 (25.0)	10 (76.9)	10 (66.6)
*Age, years*			
Mean (*SD*)	48.9 (7.5)	47.8 (8.4)	17.1 (3.5)
Range	38–57	36–66	13–23
*Age at diagnosis, ****y****ears*			
Mean (*SD*)	46.1 (7.1)	44.3 (8.9)	11.7 (6.2)
Range	35–54	33–64	1–20
*Diagnosis, n (%)*			
ALS	6 (75.0)	12 (92.3)	12 (80)
PMA	1 (12.5)	–	–
PLS	1 (12.5)	1 (7.7)	3 (20)
*Years since diagnosis, n (%)*^a^			
< 1 year	2 (25.0)	1 (7.7)	1 (6.7)
1–2 years	2 (25.0)	3 (23.1)	1 (6.7)
2–3 years	2 (25.0)	2 (15.4)	2 (13.3)
3–4 years	–	1 (7.7)	–
4–5 years	1 (12.5)	3 (23.1)	4 (26.7)
≥ 5 years	1 (12.5)	1 (7.7)	5 (33.3)
*Children in family, n (%)*^b,c^			
1	1 (12.5)	2 (15.4)	1 (6.7)
2	3 (37.5)	6 (46.2)	5 (33.3)
≥ 3	4 (50.0)	5 (38.5)	9 (60.0)
*Age children, years*			
Mean (*SD*)	15.5 (7.4)	15.3 (6.8)	–
Range	2–27	1–27	–

*Note.* ALS, amyotrophic lateral sclerosis; PLS, primary lateral sclerosis; PMA, progressive muscular atrophy. ^a^Years since diagnosis at time of interview. Adds to less than 100% for well parents and children because 2 parents with ALS deceased prior to the interview. ^b^Stepchildren were also included in the count. ^c^Adds to more than 100% for well parents due to rounding

### Procedure

This study was approved by the ethical review board at the University Medical Center Utrecht. Digital informed consent was obtained from all participants prior to participation in an interview. For children under the age of 16, also (digital) parental consent was obtained. Both parents and children were interviewed individually, either online or via telephone. Interviews were conducted in Dutch by the first author, a female postdoctoral researcher (PhD Psychology) who had previous experience with conducting interviews. She was neither known to nor involved in the care for the participating families living with ALS. The primary aim of the interviews was to shed light on opportunities for supporting families living with ALS through identifying their struggles and support needs. Prior to the start of the interview, the interviewer introduced herself as a researcher, informed participants about the purpose of the study, the duration of the interview and confidentiality and gave them the opportunity to ask any remaining questions. Interviews were conducted using semi-structured interview guides including open-ended and probing questions. Each interview began with questions about personal and illness-related characteristics. Subsequently, parents were asked about the impact of ALS on family life, how the family copes with this situation, parenting with ALS, familial communication about ALS and parental support needs whereas children were asked about their lived experiences with ALS, their involvement in the life with ALS, their current support system, and support needs. Duration of the interviews ranged from 10 to 81 min, with an average of 47 min. Interviews were audio-recorded and transcribed verbatim.

### Data analysis

Sampling, data collection, data analysis and interpretation took place iteratively. Prior to the analysis, all interview transcripts were anonymized. All information that is traceable to the participant was removed from the transcripts, for example names and places of residence. After repeated reading of the transcripts to become familiar with the narratives, interview transcripts were entered into NVivo 12 and analyzed using qualitative content analysis, that is, interview transcripts were systematically organized through assigning codes, i.e. conceptual labels consisting of words or short phrases, to relevant sentences or passages. This set of codes constitutes a coding scheme. The coding scheme was developed using both a directed and conventional approach to qualitative content analysis. In directed content analysis, codes are derived from previous theories or research, whereas in conventional content analysis, codes are derived directly from the data [16].

Using a directed approach, a first draft coding scheme was developed by the first author. This scheme comprised codes derived from previous research including but not limited to the field of ALS [3, 6–11, 13, 17–31] and served as a starting point for the content analysis. For each code, the coding scheme provided a definition and guidelines on how and when to use this code.

Throughout the coding process, the initial scheme was continuously modified, supplemented and refined based on newly emerging interview data (conventional approach) until code saturation was reached for both the parent and child interviews. All interview transcripts were coded by the first and second author, independently from one another. For each transcript, the assigned codes were compared and discrepancies were discussed until resolved. When disagreement persisted, the last author was consulted. When consensus was reached on all codes, the final coding scheme (Additional file 2) was applied by the first author to code all parent and child interview transcripts. In the final step, the codes that made up the final coding scheme were sorted into categories from narrow to broad, reflecting overarching and supporting themes. Following completion of the qualitative content analysis, quotes were selected to illustrate our findings. Quotes were translated by an independent person fluent in both English and Dutch.

To minimize researcher bias and increase trustworthiness of the research findings, we used data and researcher triangulation, peer debriefing and audit trails [32]. Data and researcher triangulation, respectively, were applied through collecting data from parents and children and discussing emerging themes with the whole research team representing diverse backgrounds (e.g., rehabilitation medicine, social work, psychology), clinical expertise in ALS (EKvR, MSK), expertise in research on caregiving/(young) caregivers (MSK, JMAVM) and expertise in qualitative research (MSS, MSK, AB). To warrant that the results were reflective of the experiences of the wider ALS patient community, peer debriefing was used. The themes identified were reviewed and deemed appropriate by two parents (i.e., parent with ALS and well parent) and one bereaved young adult who participated in a steering committee yet not in an interview. Audit trails were developed using reflexive memos and codebooks.

## Results

Three interrelated themes emerged from the interviews: (1) transformations in family life due to ALS, (2) the struggle of living with ALS, and (3) ALS families’ needs and resources for creating a supportive environment. Across those themes, we tell the stories of families living with ALS from both the parent and child perspective. An overview of themes and subthemes can be found in Table 2.

**Table 2:** Overview of themes and subthemes

Themes	Subthemes	Definitions
Transformations in family life due to ALS	Transformations in the family home	Changes in the home setting (e.g., home modifications) that impact on parents’/children’s everyday life
	Transformations in family activities	Impact of the ALS situation on family activities that are undertaken (e.g., leisure trips, holidays)
	Transformations in family roles	Changes that occur in the division of family roles (e.g., caregiving, household tasks)
	Transformations in family relationships	Changes that occur in the ways in which family members relate to, interact with and support each other
The struggle of living with ALS	Distress towards self	Extent to which the parent/child experiences distress towards the self
	Distress towards others	Extent to which the parent/child experiences distress towards other family members
	Distress towards family functioning	Extent to which the parent/child has concerns with regard to family functioning
Families’ needs and resources for creating a supportive environment	Emotional support needs	The types of support that parents/children seek to be able to deal with emotions that arise from the ALS situation and to make meaning of the ALS situation
	Psychological support needs	The types of support that parents/children seek to be able to cope with/adapt to the transformations in life imposed by the ALS situation
	Educational support needs	Education about ALS, its prognosis and treatment sought by parents/children at the time of diagnosis and throughout the course of disease
	Practical support needs	The types of practical assistance that parents/children seek to be able to reduce their burden

### Transformations in family life due to ALS

Throughout the illness trajectory, families went through different transformations. Not only families’ homes were modified to meet the needs of the parent with ALS, also family activities, roles and relationships took a different shape.

#### Transformations in the family home

Home modifications (e.g., stair lift, care unit) and the presence of healthcare professionals in the home to assist the parent with ALS with self-care impacted on the atmosphere in the home, as well as on the everyday lives of each family member.

*Our house has just been completely renovated and there is a different atmosphere*.

(Daughter, age 15, 4 siblings, lives with parents and siblings)

*You’re always trying to maintain privacy within your family and then you see that more and more other people come into your home. Not that I can't stand that, but it is very different. Privacy is important, but you also need care.* (Well parent, married, father of two late adolescents)

#### Transformations in family activities

Most families indicated undertaking similar activities as before the diagnosis yet in adjusted ways (e.g., in a mobility scooter instead of walking). For the sake of making memories, some families undertake even more activities together than before the diagnosis, especially briefly after the diagnosis when symptoms are mild and they do not know how fast the progression will be. Traveling abroad and leisure trips to for example theme parks and zoos were frequently reported as ways to spend time together as a family. As the disease progresses and it gets more and more complicated for the patient to move around, the frequency of such activities is reduced and families seek to spend time together in simple pursuits such as eating together.

*We always did that [going on vacation/trips], but we are now making even more of an effort. Celebrated lots of things together and now we’ve added outings.* (Well parent, married, mother of three young adults)

*I would really love to go mountain-walking or mudflat-walking with my family, but that’s simply not possible. And if I go on vacation, I have to take all my care with me and I am even busier than when I am at home, so that’s not an option either.* (Well parent, , married, mother of three adolescents and two young adults)

*We eat together. We went on holiday together last year. But you just notice that it is getting more and more difficult to do things together, because my father has so little energy left.* (Daughter, age 21, 4 siblings, lives with parents and siblings)

Various families were involved in community ALS activities, mainly sports events to raise money for research in ALS (e.g., Amsterdam city swim, Tour du ALS). These events were often experienced as a way to bond as a family.

*Of course, it was about that mountain and raising money [Tour du ALS], but the best thing we still have is remembering that we rented a house for everyone and apartments nearby and that for a week you just looked forward to an event like that with each other.* (Well parent, married, father of two late adolescents)

#### Transformations in family roles

Within the families, changes occurred in the division of parental tasks and responsibilities. As symptoms of the parent with ALS get worse, a shift takes place in parenting responsibilities from the parent with ALS to the well parent.

*In practice, I'm a single mother now. Only my husband is still alive and also lives at home, but the care [for the children] is simply 100 percent down to me.* (Well parent, married, mother of two children in early childhood)

In some families, the ALS situation also seemed to affect how parents fulfilled their parenting role. For instance, they became more relaxed as a parent. One parent said he felt more like a husband than a father, he experienced that his wife who had ALS needed him more than his son who did well in school and socially.

For some parents, the ALS situation encouraged them to pass on their life lessons, beliefs and values to their children, through living it or talking about it, and to preserve their memories via photography, videos or diaries for their children. For example, parents taught their children the importance of savoring small moments of happiness, to focus on the positives, to seek help, to do the things they love, to make their own choices or how to grieve.

*My wife actually started writing things down from the moment she was diagnosed. Especially for our son. Memories of his childhood, what she had experienced with him, so that he could read it later, when she was no longer around.* (Well parent, father of an adolescent, spouse passed away 3 months ago)

*The past five years have, of course, really been a roller coaster of sadness, but also of amazingly beautiful things which come your way. And we really try to show the children that it is sad but that it also opens doors. Opportunities will come your way, and people in your life who are so valuable and who give a lot of support.* (Well parent, married, mother of two children in middle childhood and early adolescence)

Besides figuring out how to maintain normalcy in daily life while living with ALS, parents are faced with the new parenting task of guiding their children through the disease trajectory. How parents fulfil this task differs across families, though their motivation is unanimously to protect their children from harm. Where the one parent involves his/her children in every treatment decision made, the other attempts to keep the children away from ALS as much as possible.

*There are so many aids and situations in the house. And we involve them [the children] with all of these. We let them figure it out, open it up, experiment with it, so that they also become familiar with it.* (Well parent, married, mother of two children in middle childhood and early adolescence)

*We are left out of many things. For example, if my father has an appointment at the hospital and he, for instance, has to have a new treatment, we are not always made aware of this. We have to ask about it ourselves. […] Personally, I would like to be more involved.* (Daughter, age 23, 4 siblings, lives with parents and siblings)

Although the well parent appears the primary caregiver in most families, children also take on a broad variety of hands-on caring activities in the domains of household tasks (e.g., vacuum cleaning, cooking, unloading the dishwasher, making the bed, shopping), sibling care (e.g., helping younger siblings with homework and transporting them to and from activities) and personal care for the parent with ALS (e.g., assisting with opening bottles, jars and yogurt/milk cartons, eating and drinking, (un)dressing, moving around in the home, changing the tube feeding bag, putting on breathing equipment).

*Put on his sunglasses, or take a tissue out of his bag for him to blow his nose. Just a 1000 things that they [children] no longer realize are actually all caring acts.* (Well parent, married, mother of two children in middle childhood and late adolescence)

*Sometimes I put him [father with ALS] to bed and then I have to rinse through his PEG probe or give him his medication or make him a cup of tea. Actually, all kinds of little things, put on his shoes, do errands for him, you name it.* (Daughter, age 23, 4 siblings, lives with parents and siblings)

*At the very end, sleeping on the couch at night. She [mother with ALS] had a ventilator and you never knew when it would come loose. Its tube came off every night for no reason. So, then somebody had to sleep on the couch at night, in the room next to hers, so that hopefully you would notice if something went wrong. And quite often it did go wrong so that was frightening.* (Son, age 19, parent with ALS passed away 3 months ago, no siblings)

#### Transformations in family relationships

Changes in parents’ and children’s roles are likely to impact on the bond between parent and child which was also reflected in the interviews. Parents and children indicated that parents with ALS become less involved physically, due to functional limitations and fatigue, yet remain involved in their children’s lives on an emotional level. Parents and children sought alternative ways to spend time together. Over the course of the disease, a transition seems to take place from out-home/active (e.g., playing sports) to in-home/passive parent and child activities (e.g., having a cup of coffee or a beer, watching television, providing support in homework activities).

*My husband used to work and was an actively involved family man. He did a lot with the girls as our eldest daughter’s volleyball coach, active at school as an auxiliary parent. A very involved, social father. And that has, of course, changed completely to being very passive.* (Well parent, married, mother of two children in middle childhood and early adolescence)

Some well parents stressed that they have less time to engage with or listen to their children due to caring responsibilities. Nonetheless, even in case of advanced ALS, parents are often able to create meaningful parent–child moments in everyday life.

*Our youngest [daughter] thinks that he [father with ALS] still does quite a lot with her*.

*Sometimes she says, Daddy we're going to play a game. And [name partner] has no hand function and can no longer speak, so what kind of game can you do? But she really feels that they are still playing Monopoly or something together. For example, he writes with his eyes on the Tobii [eye tracking controller] you have to put this card in or you have to put that card away. From her point of view, they are playing a game together. Someone else would think, what is he doing? He’s just sitting in his chair. But they get a lot of quality time out of it.* (Well parent, married, mother of two children in middle childhood and early adolescence)

*Sometimes we just try to make small things special, that Daddy helps with picking them [children] up or that they [children] can watch television with Daddy.* (Well parent, married, mother of two children in early childhood)

Parents and children in families with ALS sometimes developed different attitudes towards one another. Parents described witnessing their children becoming more independent, responsible and caring, evoking feelings of pride, awe and gratitude.

*And now they [the children] cook too. They have suddenly become super-independent, know how the washing machine works, all that sort of thing.* (Parent with ALS, married, mother of two late adolescents)

*They are extra responsible and you can see that they are considerate to their fellow human beings, because they are used to that at home.* (Well parent, married, mother of three adolescents and two young adults)

Children admired their parent with ALS for maintaining a positive attitude throughout the illness. They witnessed their parent transforming from an active, strong and independent individual to a passive, dependent and emotionally less stable person. Some began to view the parent with ALS as a patient or disabled person who is less capable to do things, while others emphasized that, in these cases, their father was still a warm and supportive parent.

*You sometimes start to see your father a bit like a patient. He often needs help with things. Your father used to take care of you and now I take care of my father.* (Daughter, age 21, 4 siblings, lives with parents and siblings)

*I also think he [father with ALS] is very nice and although he can’t do that much anymore, it is still the case that he helps and supports us by saying things.* (Daughter, age 13, lives with parents and 1 sibling)

Not only the parent–child relationship changes, but also how parents relate to, interact with and support each other. Along with the physical deterioration of the parent with ALS, the relationship between parents becomes less intimate, physically and/or emotionally, and more dependent.

*Normally, children see their parents give each other a hug or walk hand in hand or something. They don't really see that kind of thing with us.* (Well parent, married, mother of two children in early childhood)

*I would like to talk about my feelings, I want to be able to share them with [name of partner] and for [name of partner] to hear them and sort of understand. That is simply not possible now. He really can’t deal with my grief.* (Well parent, married, mother of three children in early and middle childhood)

In most families, though, the parent with ALS still has a say in parental decision making. Even when the parent with ALS experiences communication difficulties, parenting decisions, such as school choices or how to deal with behavioral issues, are usually negotiated between parents and mutually decided upon. However, the well parent is usually the one who applies the decisions.

*What he can do and what he has to offer them [the children] is, of course, very limited. But I can talk with him; he can't talk, but he can type. We can communicate about how things are with the children, for example, when it comes to upbringing and what they can and cannot do and how we deal with certain things. We can just discuss it. In that sense, he does contribute.* (Well parent, married, mother of two children in early childhood).

Some parents emphasized that all family members are in it together. Most families became closer due to the shared experience of ALS. They spend more time together as a family, are more open to each other and feel more interconnected.

*As young as they are, they are being confronted with very serious matters, and also with the fact that their father is dying. That’s really intense. Somehow, it also brings you together, because you know all too well, we have to go through it together.* (Well parent, married, mother of two children in middle childhood and early adolescence)

*Straight after the diagnosis, we said to each other, our life is well organized, we live life the way we want to live it, we don't need to change much. But I think we have learned to talk a bit more with each other, also about how you are doing and to dwell on that. I think that has been a big lesson.* (Parent with ALS, married, father of three young adults)

*You do notice that you become even closer as a family. […]. And your bond also becomes stronger, because you help each other, you see each other more often, you talk to each other more often, you actually interact more than before.* (Son, age 16, 1 sibling, lives with well parent (divorced) and sibling)

### The struggle of living with ALS

Transformations in the family home and family dynamics seem to trigger distress among families living with ALS, including anxiety, worry, frustration, sadness and guilt. As reflected in the interviews, these feelings of distress are directed at the self, other family members or the family as a whole.

#### Distress towards self

A few parents with ALS struggled with the idea of missing out on future significant moments of their children such as graduation or marriage. Well parents sometimes felt the urge to fulfil both parents’ roles, causing them distress. Others felt burdened by the thought that if anything bad happened to them that would be detrimental to the family. Whereas some parents were very confident about how they handled the ALS situation with regard to the children, others indicated feeling less competent as a caregiver for their children and insecure about how to guide their children through the illness trajectory.

*If you become disabled, if you lose functionality, that sometimes causes frustration and irritation. Then you sometimes take it out on another, and then I don't feel like such a good parent.* (Parent with ALS, married, father of two children in early and middle childhood)

*The milestones you normally reach with your family, when someone passes a final exam, […] a graduation, the idea that I would miss some of those moments and everything that comes after that. That was very confronting and sad, not just for me, for all of us.* (Parent with ALS, married, father of three young adults)

*Since then, she has always been the girl with the father who is ill or who will soon pass away, and that is something you will carry with you for the rest of your life and which you, as a surviving parent, can only properly guide once. I do hope that I can just do well.* (Well parent, married, mother of two children in early childhood)

#### Distress towards others

Both the parent with ALS and the well parent worry about the impact of ALS on their children’s present and future lives. They worry that their children do not receive enough attention, love and support, will miss out on being kids, are burdened as young caregivers, will miss a father/mother figure in their life and that they will carry this painful experience with them the rest of their lives causing them harm. One mother worried that her child might get bullied in school because her father with ALS is in a wheelchair.

*They [the children] are, of course, young caregivers. They can’t escape it. We are very conscious of that, but it is also a very gray area. Because we do not want them to be caregivers and no longer children.* (Parent with ALS, married, father of two children in early and middle childhood)

*We have also had an ambulance here because [name partner] choked and we were afraid he would suffocate. That also affects a child.* (Well parent, married, mother of one adolescent and two young adults)

In a few cases of familial ALS, parents worried that their children someday might have to go through the same painful journey they are going through now and may not be able to live the lives they want.

As parents’ attention mostly turned to how the children in the home were coping, likewise children were often more concerned with their parents’ well-being than their own well-being. They worry that the parent with ALS might be in pain, that something happens to them when they are not around, and find it difficult to witness their parent’s physical and emotional hardships.

*I always keep an eye on my phone worried that Daddy will fall again and that no one finds him. It’s on your mind every day that nothing will happen to him.* (Daughter, age 19, 2 siblings, lives with well parent (divorced) and 1 sibling)

Some children indicated that they worried rather about the well parent who had to keep the family running than about the parent diagnosed with ALS.

*Certainly lately I’ve been constantly worried about my father. He is quite old and it is quite difficult to see someone slowly die. And you have to take care of her. It was tough for him.* (Son, age 19, parent with ALS passed away 3 months ago, no siblings)

#### Distress towards family functioning

Some well parents and children had concerns about how the (bereaved) family would function in the end-of-life phase or the period following the death of the parent with ALS.

*And later when I’ll be alone with three adolescents who are sad. Then I think, oh God, what's that going to be like?* (Well parent, married, mother of three children in early and middle childhood)

*When he gets worse and we maybe have to watch over him, will we be able to manage as a family? I sometimes think about that.* (Daughter, age 23, 4 siblings, lives with parents and siblings)

### ALS families’ needs and resources for creating a supportive environment

The ALS situation prompted emotional, psychological, educational and practical needs in the families that took part in our study. To meet those needs, families use resources at family, social environment and/or healthcare level.

#### Emotional support needs

Parents’ and children’s emotional support needs relate to the types of support they seek to be able to deal with the emotions that arise from the ALS situation and to make meaning of the ALS situation. This includes the need to talk about the ALS situation and how one experiences it as well as the need to receive compassion and positive encouragement from others. Moreover, some parents exhibited a need for emotional empowerment for them to be able to better recognize emotions in their children and help them cope with their emotions as well as to better manage their own emotions in front of their child and remain emotionally available to their child.

*As a parent, what do you share with your children about your own feelings? You want to be a pillar of strength, but as a parent you also have feelings. These are things that go through your mind.* (Well parent, married, mother of three young adults)

*Especially my mother, she is sometimes rather more concerned with herself and how she can handle this situation than asking about what it is like for us as children.* (Daughter, age 23, 4 siblings, lives with parents and siblings)

Children indicated that parents can support them by attending to how they are experiencing the ALS situation and preparing them for major changes. For some children, though, the emotional support need was not so much in talking about the ALS situation on a regular basis, but rather in others “being there” for them.

Parents provide emotional support to their children but sometimes it is also vice versa.

*Of course, my mother had to tell a number of people [about the diagnosis]. I had to help with that every now and then, as she couldn’t express herself, because of the crying more than because of the ALS.* (Son, age 19, parent with ALS passed away 3 months ago, no siblings)

Outside the family, both parents and children receive emotional support mainly from relatives and friends. They support ALS families not only through listening to them, but also through engaging in family-initiated ALS activities. Some parents and children preferred sharing experiences with peers in a similar situation, though only a small number of parents and children managed to establish peer contact, usually via personal connections. In some cases, emotional support was sought from a professional such as a psychologist, social worker, practice nurse or school counsellor.

*I talk to a psychologist about it, because I realize that I am bottling it up. I think a psychologist is really good for me to express everything.* (Daughter, age 19, 2 siblings, lives with well parent and 1 sibling)

In one family, religion gave family members the strength to deal with the ALS situation.

*I can draw on my faith, every day I get the strength for it. Sometimes I think a normal person would have fallen apart long ago.* (Well parent, married, mother of three adolescents and two young adults)

#### Psychological support needs

Psychological support needs relate to the types of support that parents and children seek to be able to cope with transformations in family life. For dealing with the distress imposed on them by the inevitable transformations in family life, both parents and children used a range of coping strategies including acceptance of the (immutability of the) ALS situation, positive refocusing (i.e., directing attention towards the positives in life), mindfulness (i.e., living in the present rather than thinking about the future), savoring simple everyday moments, humor, and distracting oneself.

Some parents draw on previous education (e.g., in psychology, pedagogy or education) and/or experiences with ALS or other illnesses in their social network, giving them tools on how to handle the ALS situation.

*We've already had a time when we had to share a bad diagnosis with the girls. Back then, they were obviously much younger, but that was quite a lesson for us that has also helped us now.* (Parent with ALS, married, father of three young adults)

Other parents require psychological support to master the challenges of living with ALS. Via healthcare professionals (e.g., psychologist), books and Facebook groups, parents seek advice in terms of how to raise and support their children in the context of ALS. Questions relate to how and when to break the news of the parent’s diagnosis, how to explain ALS, how to involve children throughout the illness process and opportunities for parenting support.

*You want to protect them [children] and if you can't protect them you want to help them get through it as well as possible. And that was quite a search: How do you do that? […] I had a lot of questions.* (Well parent, mother of two adolescents, spouse passed away 8 years ago)

*It's not about which book we can write for the kids to understand ALS. It’s about which book we can write so that parents can explain to their child that they have ALS. It's about supporting the system and not just children. Because the most important book for children is their parent. So that’s who we have to support.* (Parent with ALS, married, father of two children in early and middle childhood)

Parents not only seek psychological support for themselves but also for their children, to help them cope with having a parent with ALS.

*I also made some calls to authorities to ask what they offer for children with a terminally ill parent. It is mostly about grief counseling, but that's not what I'm looking for.*

*[…] I am looking for things about how to cope with an ill parent.* (Well parent, divorced, mother of two adolescents)

A few children indicated having received and benefitted from psychological support provided by a professional. Others were reluctant to or felt uncomfortable disclosing their feelings and experiences to a psychologist or social worker.

Multiple parents indicated that they would like their children to build a relationship with a social worker, psychologist or other healthcare provider, to lower the threshold to seek help when in need.

*I would have liked it if they [children] had been able to build a bond with a psychologist or a child therapist. Now I had to play therapist myself. […] Even if you only allow children to become familiar with someone. That if they are in need, they have someone to fall back on.* (Well parent, mother of two adolescents, spouse passed away 8 years ago)

A number of parents and children pointed out that ALS care should be centered on the family rather than the patient only, empowering family members to deal with the ALS situation together and maintain a well-functioning family. In this regard, family conversations with a doctor or social worker were considered a valuable manner to improve family communication, family relationships and overall family functioning in the context of ALS.

*I think some kind of family meeting with some kind of professional would help us a lot.*

*To be able to talk as a family with someone outside the situation about how everything is going and what we encounter and where it goes wrong.* (Daughter, age 21, 4 siblings, lives with parents and siblings)

#### Educational support needs

Four types of educational needs emerged from the interviews: education for parents, children, schools and friends/relatives. Parents seek objective information about ALS, its prognosis and treatment as well as experiential stories from other parents living with ALS, so as to prepare themselves as well as their children for what is coming. Children seek similar information as their parents. They want to know what ALS is, how it progresses, what treatment entails and like to read how other children with a parent with ALS experienced it so that they know what to expect of the future.

*It is good to know what exactly it [ALS] entails, what kind of prognosis there is, the life expectancy and so on. Then you can also be better prepared for what might come.*

(Son, age 22, 2 siblings, parents divorced, lives on his own)

*I would like to read how others feel about it and how they cope and maybe some tips on how to deal with certain things, illness stories.* (Daughter, age 21, 4 siblings, lives with parents and siblings)

A daughter with a father with ALS mentioned she lacked information on cognitive changes in ALS.

*I notice that his mind is also changing, which makes sense, but there are also things changing that he is not aware of [...] and that he kind of tells things that are not true at all, but are true in his eyes. I did not expect that, so that's a bit surprising. They hadn’t told me that he would also say things that are not true.* (Daughter, age 18, 1 sibling, lives with well-parent (divorced) and sibling)

In addition, some children exhibited a need for education and practical training in ALS caregiving, for instance how to use medical equipment (e.g., stair lift, stomach tube, respiration) and how to handle life-threatening situations (e.g., when parent is choking).

*That he [father with ALS] has the feeling that he is choking, that does happen regularly.*

*Then there is some mucus in the throat, he no longer has the strength to cough it up and gets kind of an attack. There is not much you can do about it. You just have to wait for him to come round. As long as he is making a noise, then it’s all right and once he goes quiet, then I think you should call 112 [emergency number].* (Daughter, age 21, 4 siblings, lives with parents and siblings)

Where parents were educated about ALS primarily through ALS care professionals during hospital visits, children were primarily educated by their parents. Furthermore, they consult the Internet and/or books or brochures aimed at children (alone or together with a parent). Those who provided care to their parent with ALS were instructed by their parent and/or ALS/home care professionals on how to perform everyday caregiving tasks.

Multiple parents argued that schools can play an important role in guiding children through the ALS situation and supporting their development. In general, parents informed their children’s school about their home situation. In some cases, children informed their teacher/mentor and/or class at school themselves. Although most parents and children felt supported by the school, a few parents suggested to develop information written for schools which parents can use to educate teachers/school staff about ALS so as to create a supportive school environment for their children.

*I think it might also be a good idea to develop something for schools and daycare centers to explain what ALS is and how to tell certain age groups about it.* (Well parent, married, mother of two children in early childhood)

Some parents and children in families with ALS struggled with how to deal with incomprehension from people in their social environment, evoking a need for materials that can be used by families to educate their social network about ALS.

*The biggest problem the children encountered was lack of understanding among their peers.* (Well parent, mother of two adolescents, spouse passed away 8 years ago)

*When they asked my daughter "What does your mother have?", she forwarded that video [about ALS]. So that she didn't have to explain.* (Parent with ALS, married, mother of two late adolescents)

#### Practical support needs

Some practical support needs came up in the interviews. Well parents need others to reduce their caregiving workload through assisting with household tasks (e.g., cooking a meal) or spending time with the parent with ALS, so that they can have more quality time with their children. Most families on a regular basis received domestic aid and help with personal care of the parent with ALS from home care professionals. Often, friends and relatives also help out. Several parents also indicated receiving some kind of parenting support, in terms of close relatives (e.g., the children’s grandparents) or friends taking their children to activities or babysitting.

*Having someone do the laundry and ironing or folding or cleaning the outside of the windows, that helps me more. Then I can give more attention to the children, because my household chores and my husband's illness take all my time.* (Well parent, married, mother of three adolescents and two young adults)

## Discussion

This study adds to the existing ALS literature base through empirically exploring what parents and children in ALS families need to master life with ALS. The findings demonstrate that parental diagnosis of ALS transforms family life in terms of activities, roles and relationships. The distress imposed on the family members evokes four types of support needs, namely (1) emotional needs, relating to sharing experiences; (2) psychological needs, relating to mastering the challenges of life with ALS; (3) educational needs, relating to ALS knowledge and caregiver training; and (4) practical support needs, relating to reducing caregiver burden. To meet these needs, families use a range of resources at family-, social environment- and healthcare-level.

Our findings illustrate that not only the parent with ALS but the whole family transforms along with changes in family dynamics imposed by the symptoms associated with ALS. In the current study, emphasis is on the negative transformations that occur within families. Despite that ALS brings many losses not only for the parent with ALS but also for the well parent and their children, we recognize that the ALS experience may also bring about positive transformations, such as personal growth, more connectedness across family members and the shared experience of memorable trips or (ALS) activities. Being able to see these positive experiences may strengthen resilience and help family members to maintain psychological quality of life despite that their physical quality of life remains impaired [33, 34]. In the present study, many interviewees indeed reported focusing on the positives in life, savoring simple everyday moments, and being more mindful which helped them to maintain well-being. Other coping strategies that were reported as being helpful for minimizing distress by our sample included acceptance, distracting oneself, using humor, acquiring information about ALS, seeking social support, maintaining daily routines and making new positive memories. Despite their struggles, most parents and children in our sample ultimately showed being resilient. Those families who are less resilient though may benefit from psychological interventions aimed at fostering adaptive coping strategies which, in turn, may buffer against internalizing problems such as depression and anxiety [34]. Given that ALS affects the everyday lives of all family members, these kinds of interventions should be targeted at people living with ALS as well as their spouse and child carers.

In recent years, the need for support targeted at children of chronically ill parents is increasingly recognized by researchers and practitioners (e.g., [17, 35]). A first step towards adequately supporting children of parents with ALS is empowering parents to support their children by increasing their knowledge, skills and parental sense of competence. Parental empowerment will ultimately have a beneficial impact on children’s psychosocial functioning, because when parents are less distressed so are their children [22, 25, 30]. Considering that children of parents with ALS are already vulnerable in multiple respects [5, 8] and that disrupted or compromised parenting due to a parent’s ALS and associated care needs may aggravate the vulnerability of children at present and in later life [36], it is crucial to support parents with ALS and their co-parents in their parenting role.

In addition to the emotional and educational support needs highlighted in previous research among ALS patients and/or their family caregivers [13, 18, 24], we identified some psychological and practical support needs beyond the normal care needs which specifically pertain to parenting and child-rearing. In the light of previous work demonstrating that open and supportive family communication protects against child maladjustment in families with a parent with a life-limiting illness (e.g., [20, 21, 30, 37]), a parental need that deserves special attention and is currently insufficiently met is the need for guidance on how to communicate about ALS with children.

Our findings demonstrate that parents with ALS generally turn to people in their social network, most notably close relatives and friends, to ask for emotional and practical support, whereas they seek information and psychological guidance from healthcare professionals. ALS care teams may thus play an important role in the psychological and educational empowerment of parents living with ALS. The needs identified in the interviews translate to a number of opportunities for ALS care professionals to support families with ALS. They may want to educate families about ALS, involve families’ social network in supporting them, teach parents how to talk about ALS with their children, how to recognize emotional processing difficulties in their child, how to deal with their own and children’s emotions, and how to involve children in the illness trajectory without burdening them, and make parents aware of opportunities for parenting support.

While it becomes clear that parents living with ALS need support in meeting the needs of their children and that ALS care professionals, such as social workers, may provide this kind of support to parents, we also realize that these professionals may need to be supported themselves in order to adequately and timely identify these parental needs and to provide optimal parental support [30]. For example, whereas ALS care professionals may be expected to possess sufficient ALS-related knowledge, they may not feel prepared to engage parents and children in a dialogue about parental needs, children’s needs or family functioning [38]. For those who frequently have to deal with families, it may therefore be helpful to receive education regarding family-oriented care and processes of grief and loss among different age groups, training in conversation techniques, tools for screening family dysfunction, and information to hand out to parents and/or children. We encourage the development of guidelines and tools to facilitate ALS care professionals to support parents in their parenting roles.

While parental empowerment makes sense as a first step towards improving support for families with ALS, efforts can be made to reach out to the children living in these families. We identified a number of protective variables that help children to successfully adapt to the ALS situation, including a positive outlook on life, knowledge about ALS (e.g., symptoms, prognosis, medical equipment), supportive communication about ALS within and outside the family, and a good balance between caring for one’s parent and living one’s own life. Support programs aimed at children with a parent with ALS may benefit from a focus on these topics.

Where the possibilities for ALS care professionals to support children in families with ALS are limited due to time constraints and children being in school during the week, they may offer some sort of educational support through providing children (via their parents) with age-specific written information about ALS. Schools, where children spend a great deal of their time, may play an important role in offering emotional support and encouragement to children, e.g., through school counseling. To do so, it is crucial that school teachers, mentors, counselors or nurses are adequately educated about how to support students with a parent with a progressive and terminal illness such as ALS. Collaboration between ALS care professionals and schools may contribute towards better mental health and school achievements among children living with a parent with ALS.

Strikingly, the needs reported by ALS families were highly similar to the needs reported by families with other life-limiting illnesses, such as Huntington’s disease, cancer or multiple sclerosis [19, 20, 26–28, 37]. It seems that except for educational needs the support needs of families living with ALS are not so much disease-specific yet may be more urgent compared to other illnesses given the very progressive nature of ALS. Due to the dynamic and unpredictable course of ALS, families’ support needs are also dynamic rather than static. Families’ needs change continuously, driven by transformations in family functioning that occur as the result of exacerbations of existing symptoms and the onset of new symptoms in the parent with ALS. Our findings imply that the types of needs that arise among ALS families are similar yet of different priorities. Which need has priority in a family depends among others on familial resources and values, children’s developmental stage, the time since diagnosis, the current stage of the illness (symptoms), and how rapidly the disease is progressing. Tailoring support to the changing needs of families facing ALS requires awareness, skill and experience on the part of healthcare professionals serving those families. This further emphasizes the necessity of educating and training ALS care professionals in how to support families with ALS.

### Limitations

Our findings should be interpreted in the light of some limitations. First, our convenience sampling strategy may have resulted in selection bias. Indeed, our subsamples were rather homogeneous in terms of demographics (e.g., gender, education). Mothers with ALS and male well parents were underrepresented, although it should be noted that similar themes emerged across fathers and mothers in families with ALS. The majority of families represented in this study were traditional native Dutch families with a father, mother and one or more children. Therefore, our findings are not reflective of the experiences and perspectives of among others minority, single-parent and same-sex families. Despite previous research suggesting that a considerable proportion of ALS patients exhibit some form of behavioral and/or cognitive impairment [39], remarkably, none of the parents and children we interviewed indicated struggling with behavioral impairments such as apathy or behavioral disinhibition. Additionally, we noticed that a considerable proportion of parents were educated in the field of healthcare, pedagogy or education, which may be considered an important resource for coping with ALS. As such, parental support needs may have been underestimated.

Second, our findings may have been influenced by the COVID-19 pandemic. Due to COVID-19, the lives of families with ALS were disrupted even more profoundly than by ALS alone leading to additional vulnerabilities and needs. Social distancing and isolation limited access to social resources and may have caused some families to experience more unmet needs. In this respect, it should be noted that we did not code sentences or passages explicitly pertaining to needs or transformations related to COVID-19 pandemic regulations/restrictions. Third, although some parents and children expressed concerns with regard to the period following the death of the parent with ALS, the primary focus in the interviews was on parental and children’s support needs during the illness period. We did not address support needs following parental loss. Despite aforementioned limitations, the extensive qualitative data gathered in our interview study has led to rich and informative insights on the lived experiences and support needs of families living with ALS from the perspectives of parents with ALS, well parents and children.

### Future research

Additional research is required to examine variations in ALS families’ support needs through time as well as following the death of the parent with ALS, and the impact of gender, cultural/familial variables (e.g., religion, family structure) and illness characteristics (e.g., ALS vs. PMA/PLS, spinal vs. bulbar onset, sporadic vs. familial ALS, with vs. without frontotemporal dementia) on these needs.

Of particular concern are behavioral impairments which are a predictor of family caregivers’ burden [40] which, in turn, is expected to negatively impact on family functioning. Behavioral impairments may impart higher demands on parents in their role of “caregiver”, leaving less time to fulfil parental responsibilities. Future research may shed light on the effects of behavioral impairments on the parental role of both the parent with ALS and the well parent.

Moreover, how families talk about ALS remains an important topic for future research given its profound impact on how particularly children make meaning of and cope with ALS.

### Conclusions and practice implications

This study confirms that when a parent is diagnosed with ALS, this has a profound impact on how families function and interact with each other and points to different types of support that may help parents and children to adapt to their new roles and lives. Through identifying emotional, psychological, educational and practical support needs of families living with ALS, our findings address an important knowledge gap in the field of ALS. These needs as well as the lessons learned from other medical fields serving palliative or chronically ill populations facing similar needs may guide the development of support services for families with ALS. Support should be tailored to each family’s needs, considering illness stage and progression, children’s developmental stage, familial beliefs and values, and the support network surrounding the family. Here lies an important task and responsibility for ALS care professionals who may empower families with ALS through educating family members about ALS, promoting familial communication about ALS and addressing the challenges of family life with ALS. Yet, in order to do so, they may need to be empowered themselves to adopt a more family-oriented approach to care. In the absence of a cure for ALS, all efforts that may contribute towards improving psychological adaptation and quality of life among families with ALS are strongly warranted.

## Supplementary Information

**Additional file 1**. Consolidated criteria for reporting qualitative studies (COREQ): 32-item checklist.

**Additional file 2**. Coding scheme.

## Data Availability

The interview data collected and analyzed during the current study are available from the corresponding author on reasonable request.

## Abbreviations

ALS: Amyotrophic lateral sclerosis
COREQ: Consolidated criteria for reporting qualitative research
PLS: Primary lateral sclerosis
PMA: Progressive muscular atrophy
