# Supplementary material for: Parental and child adjustment to amyotrophic lateral sclerosis: transformations, struggles and needs
Source: BMC Psychol. 2022 Mar 17;10:72. doi: 10.1186/s40359-022-00780-1 (PMC8929294; doi:10.1186/s40359-022-00780-1)
Supplement: Supplementary file 1 — Additional file 1. Consolidated criteria for reporting qualitative studies (COREQ): 32-item checklist. [file 40359_2022_780_MOESM1_ESM.pdf]

Consolidated criteria for reporting qualitative studies (COREQ): 32-item checklist

Developed from: Tong A, Sainsbury P, Craig J. Consolidated criteria for reporting qualitative research (COREQ): a 32-item checklist for interviews and focus groups. *International Journal for Quality in Health Care*. 2007. Volume 19, Number 6: pp. 349 – 357

**No. Item**	**Guide questions/description**	**Reported on Page #**
**Domain 1: Research team and reflexivity**		
Personal Characteristics		
1. Interviewer/facilitator	Which author/s conducted the interview or focus group?	Methods, page 7
2. Credentials	What were the researcher's credentials? E.g. PhD, MD	Methods, page 7
3. Occupation	What was their occupation at the time of the study?	Methods, page 7
4. Gender	Was the researcher male or female?	Methods, page 7
5. Experience and training	What experience or training did the researcher have?	Methods, page 7
Relationship with participants		

6. Relationship established	Was a relationship established prior to study commencement?	N/A
7. Participant knowledge of the interviewer	What did the participants know about the researcher? e.g. personal goals, reasons for doing the research	Methods, page 7
8. Interviewer characteristics	What characteristics were reported about the inter viewer/facilitator? e.g. Bias, assumptions, reasons and interests in the research topic	Methods, page 7
**Domain 2: study design**		
Theoretical framework		
9. Methodological orientation and Theory	What methodological orientation was stated to underpin the study? e.g. grounded theory, discourse analysis, ethnography, phenomenology, content analysis	Methods, page 7
Participant selection		
10. Sampling	How were participants selected? e.g. purposive, convenience, consecutive, snowball	Methods, pages 4-5
11. Method of approach	How were participants approached? e.g. face-to-face, telephone, mail, email	Methods, page 5
12. Sample size	How many participants were in the study?	Methods, page 5
13. Non-participation	How many people refused to participate or	N/A

	dropped out? Reasons?	
Setting		
14. Setting of data collection	Where was the data collected? e.g. home, clinic, workplace	Methods, page 7
15. Presence of non-participants	Was anyone else present besides the participants and researchers?	N/A
16. Description of sample	What are the important characteristics of the sample? e.g. demographic data, date	Methods, pages 5-6 (Table 1)
Data collection		
17. Interview guide	Were questions, prompts, guides provided by the authors? Was it pilot tested?	Methods, page 7
18. Repeat interviews	Were repeat interviews carried out? If yes, how many?	N/A
19. Audio/visual recording	Did the research use audio or visual recording to collect the data?	Methods, page 7
20. Field notes	Were field notes made during and/or after the interview or focus group?	Methods, page 9
21. Duration	What was the duration of the inter views or focus group?	Methods, page 7
22. Data saturation	Was data saturation discussed?	Methods, page 8
23. Transcripts returned	Were transcripts returned to participants for comment and/or correction?	N/A
**Domain 3: analysis and**		

**findings**		
Data analysis		
24. Number of data coders	How many data coders coded the data?	Methods, page 8
25. Description of the coding tree	Did authors provide a description of the coding tree?	Additional file 2
26. Derivation of themes	Were themes identified in advance or derived from the data?	Methods, pages 7-8
27. Software	What software, if applicable, was used to manage the data?	Methods, page 7
28. Participant checking	Did participants provide feedback on the findings?	N/A
Reporting		
29. Quotations presented	Were participant quotations presented to illustrate the themes/findings? Was each quotation identified? e.g. participant number	Results, pages 11-27
30. Data and findings consistent	Was there consistency between the data presented and the findings?	Results, pages 9-27
31. Clarity of major themes	Were major themes clearly presented in the findings?	Results, pages 9-10
32. Clarity of minor themes	Is there a description of diverse cases or discussion of minor themes?	Results, pages 10-27
