# Supplementary material for: Parental and child adjustment to amyotrophic lateral sclerosis: transformations, struggles and needs
Source: BMC Psychol. 2022 Mar 17;10:72. doi: 10.1186/s40359-022-00780-1 (PMC8929294; doi:10.1186/s40359-022-00780-1)
Supplement: Supplementary file 2 — Additional file 2. Coding scheme. [file 40359_2022_780_MOESM2_ESM.docx]

**Coding scheme**

| Themes | Primary codes | Secondary codes | Tertiary codes | Definitions |
| --- | --- | --- | --- | --- |
| Transformations in family life due to ALS | Family home |  |  | Extent to which the family has to deal with changes in the home setting that impact the everyday life of the child, parent or family, e.g. home modifications (e.g. stair lift), care unit in the garden, caregivers walking in and out. These changes may impact the atmosphere in the home as well as family members’ sense of security and privacy. |
|  | Family activities |  |  | Impact of ALS situation on family activities that are undertaken (e.g., leisure trips, holidays). This involves activities with the family as a whole, thus not one-on-one parent-child activities. |
|  |  | Making memories |  | Parents undertake special activities with their families to create positive memories. |
|  |  | ALS activities |  | This relates to involvement in ALS at community level. The family is involved in ALS-related activities, including participation in research or events for collecting money for ALS (e.g. Lenteloop, Amsterdam city swim, Tour du ALS), or activities to create awareness about ALS. |
|  | Family roles | Youth caring activities |  | Children become involved in hands-on caring activities, i.e., the child/adolescent takes on, household tasks, sibling care and/or personal care for the ill parent such as assistance with walking, eating, toileting and administering medications. |
|  |  | Parental roles |  | Changes that occur in the division of parenting tasks/responsibilities and how parents fulfil their parenting role. Due to ALS, parents have to find new ways to fulfil “old” parental roles and they encounter new parental roles such as guiding their children through the ALS situation. Parental roles relate to taking care of children’s physical and psychological well-being. This includes e.g. meeting children’s biological needs (food, shelter, clothing, medical and dental care), providing a safe, nurturing and supportive environment that allows for a healthy development (daily routines, leisure and education), but also offering guidance and direction in the process of personal development and growth (e.g. helping them develop healthy relationships and coping strategies). Due to the ALS situation, parents may for example be unable to attend school events or transport their children to and from activities/school. |
|  |  |  | Parental identity | Extent to which the parent identifies with and is committed to his/her parent roles (e.g. putting the children first). |
|  |  |  | Legacy leaving | This relates to the non-material legacy that parents leave to their children. The parent passes on elements of themselves, such as identity, values, beliefs, and family and cultural history. For example, parents teach their children what’s important in life and pas along their life lessons. Parents can leave a legacy through living it (be an example), keeping a diary, sharing family stories with their children or making videos/a memory box. |
|  |  |  | Search for normalcy | The parent tries to add normalcy to their children’s lives, e.g. through maintaining daily routines. |
|  |  |  | Involving children in disease trajectory | Extent to which parents involve their children throughout the disease trajectory. This relates to e.g. openness with regard to the disease, involving children in treatment decision making, involving children in care but also setting boundaries with regard to children’s caring activities. |
|  | Family relationships |  |  | (Changes in) the ways in which family members relate to, interact with and support each other. This relates to the family as a whole. E.g. family members spend more time together, share feelings, feel more connected to one another. |
|  |  | Parent-child relationship |  | Changes in the bond/contact between child/adolescent and ill/well parent, e.g. due to parentification. |
|  |  |  | Parental involvement | Extent to which the parent emotionally and physically participates in the child’s life (e.g. school, hobbies). This relates to parent-child activities that may impact the bond between parent and child. Due to functional limitations (patient) or caring responsibilities (partner) parents may have less time to engage with or listen to their children. Examples of parental involvement include helping children with homework, supporting children in sports activities, undertaking activities together (watching a movie, playing soccer). |
|  |  |  | Attitude towards parent | (Changes in) attitude of the child/adolescent towards the ill/well parent in the context of ALS. |
|  |  |  | Attitude towards child | (Changes in) the parent’s attitude towards the child in the context of ALS. For example, admiration for how the child handles the ALS situation. |
|  |  | Parent-parent relationship |  | Changes in the parent-parent relationship due to e.g. role changes. This relates to (changes in) the ways in which parents relate to, interact/co-operate with and support each other in the context of ALS. |
| The struggle of living with ALS | ALS-related distress |  |  | Extent to which parents/children experience distress due to the ALS situation or struggle living with ALS. Distress may be directed at the self, another family member or the family as a whole. |
|  |  | Distress towards self |  | Extent to which family members experience distress towards the self. Parents may e.g. fear missing out on future significant moments of one’s children such as graduation or marriage, struggle with their new parental roles, and feel less competent as a parent.  Children may fear the prospect of a future without a father/mother to guide/support them and experience difficulties with their new roles as youth caregiver. |
|  |  |  | Parental sense of competence | Extent to which the parent perceives him/herself or the partner/co-parent as a competent and effective caregiver in the ALS situation. For instance, due to ALS, parenting strategies for handling behavioural issues may become less effective, because parents do not have the time or strength to deal with it. |
|  |  | Distress towards others |  | Extent to which family members experience distress towards other family members. A child/adolescent may e.g. have worries about the parent who is caregiver/will be left behind or worries about the ill parent who is admitted to the hospital. A parent may have concerns about the impact of the ALS situation on the children. This includes for example: worries about the future of one’s children, worries that children are missing out on being kids, worries that children do not receive enough attention/support, feelings of guilt, worry and sadness over their children’s burden as a young caregiver and the fact that their children are going to lose a parent. |
|  |  | Distress towards family functioning |  | Extent to which the parent/child has concerns with regard to family functioning, e.g. concerns about changes in family dynamics or worries about how the (remaining) family will function when the ill parent has passed away. |
| Families’ needs and resources for creating a supportive environment | Emotional support |  |  | Care and support needs on an emotional level in the context of ALS. This relates to abilities to deal with emotions that arise from the ALS situation and make meaning of the ALS situation. |
|  |  | Emotional support for children |  | This relates to children’s need to talk about the parent’s illness, prognosis and treatment and/or one’s own experiences and losses, children’s need to be treated as normal, children’s need for others to “be there” for them in ways that are not talk-based (physically or virtually) and children’s need to feel heard and understood in the ALS situation. |
|  |  | Emotional support for parents |  | This relates to parents’ need to talk about their own parental experiences and losses as well as parents’ need for support/guidance in recognizing emotions in their child, talking with their child about emotions, helping their child cope with emotions or in managing their own emotions in front of their child and remaining emotionally available to their child. |
|  | Practical support |  |  | Need for practical assistance that reduces the burden for parents and/or children. |
|  |  | Assistance with caring activities |  | Need for others (e.g. relatives, friends, professional caregivers) to reduce the caregiving workload for the well parent and/or the child through helping with household tasks or spending time with the ill parent/children. |
|  |  | Assistance with parenting |  | Parents’ need for assistance with executing parenting tasks such as picking up kids from school or taking them to activities. |
|  | Educational support |  |  | Need to be educated about ALS, its prognosis and treatment at the time of diagnosis and throughout the course of disease. This involves acquiring particular knowledge or skills. People may acquire knowledge through seeking objective information about e.g. the illness or medical devices/procedures and/or subjective information (e.g. experiential stories). |
|  |  | ALS education for parents |  | This relates to the type of education that parents seek for themselves to be able to e.g. explain ALS to their children and prepare their children for what is coming. |
|  |  | ALS education for children |  | This relates to the type of educational support (e.g. knowledge about ALS, youth caregiver training) that children seek for themselves or that parents seek for their children. Parents may desire information about ALS written for children to explain ALS to their children. Youth caregivers may also need advice, education, and practical training in providing everyday care to the ill parent. |
|  |  | ALS education for school |  | Need for educational information written for teachers/school staff which explains ALS. Parents can use this to inform their children’s school so as to create a supportive school environment for their children. |
|  |  | ALS education for relatives and friends |  | Need for educational information written for relatives and friends on how to support families with ALS so as to create a supportive environment. |
|  | Psychological support |  |  | Psychological care and support needs in the context of ALS. This relates to abilities to master the challenges of living with ALS. |
|  |  | Child empowerment |  | The child/adolescent needs psychological support or guidance to cope with the ALS situation and improve mental health. |
|  |  | Parent empowerment |  | The parent needs to be strengthened in one’s parenting role. This involves e.g. positive reinforcement of parental identity, enhancing parental confidence and sense of competence, learning effective parenting strategies, and improving parent-child communication about ALS. |
|  |  | Family empowerment |  | The family needs to be empowered to deal with the ALS situation together and to improve family communication and family relationships. |
|  | Family resources |  |  | At the family-level, personal attributes and coping skills as well as previous experiences may aid parents and children in adapting to the ALS situation. |
|  |  | Personality |  | Personality characteristics that impact how parents/children deal with the ALS situation. |
|  |  | Religion |  | Support from religious beliefs and/or participation in religious activities (e.g. praying). |
|  |  | Coping strategies |  | This includes strategies that parents/children in families with ALS use to cope with changes in family life due to ALS. This relates to how parents cope with ALS-related distress (in relation to the children), how children cope with ALS-related distress and how they cope with ALS-related distress as a family. Coping strategies may be initiated by the parent, the child or both. Coping strategies may include using humour, normalising, focusing on the positive things and avoidance. Seeking help/support from one’s social network may also be considered a coping strategy yet is treated as a separate code. |
|  |  | Previous illness experiences |  | Previous experiences with illness (e.g. partner, other family member or friend was seriously ill) and/or healthcare services that offer parents/children guidance on how to handle the ALS situation. |
|  |  | Education and training |  | A relevant educational background, e.g. education in psychology, pedagogy, healthcare or education, that aids parents/children in e.g. ALS caregiving or communicating about ALS. |
|  | Social environment resources |  |  | Having (the need for) a social network to turn to when experiencing difficulties handling the ALS situation. |
|  |  | Relative support |  | Need for relatives to be involved in the ALS situation and offer support and positive encouragement with regard to the children. |
|  |  | Friend support |  | Need for friends, acquaintances or neighbours to be involved in the ALS situation and offer support and positive encouragement with regard to the children. |
|  |  | Teacher/school support |  | Need for teachers to be aware of/involved in the ALS situation and offer support in guiding the child through the ALS situation. |
|  |  | Work support |  | Parents’ or children’s need for their employer/colleagues to support them, e.g. one’s employer may offer practical support through making certain work arrangements (e.g. flexible working hours) allowing the parent/child to spend more time with their family. |
|  |  | Peer support |  | Need for support from peers/fellow parents living with ALS, either face-to-face or online (e.g. via a Facebook group). This also includes support from the ALS community through ALS events. |
|  |  | Other support |  | This includes support from e.g. a domestic help/housekeeper. |
|  | Healthcare resources |  |  | At the healthcare-level, we distinguish between in-person and non-person healthcare resources. |
|  |  | In-person healthcare resources |  | This relates to support from ALS care professionals or other healthcare professionals, counsellors or coaches. |
|  |  | Non-person healthcare resources |  | Non-person healthcare resources may include e.g. written educational materials such as books and brochures received from the doctor and online information platforms for parents/children living with ALS. |
